# Effect of bioaugmentation on digestate metal concentrations in anaerobic digestion of sewage sludge

**DOI:** 10.1371/journal.pone.0235508

**Published:** 2020-07-02

**Authors:** Agnieszka Montusiewicz, Aleksandra Szaja, Iwona Musielewicz, Agnieszka Cydzik-Kwiatkowska, Magdalena Lebiocka

**Affiliations:** 1 Lublin University of Technology, Faculty of Environmental Engineering, Lublin, Poland; 2 University of Warmia and Mazury in Olsztyn, Faculty of Environmental Sciences, Olsztyn, Poland; Indian Institute of Technology Patna, INDIA

## Abstract

This study examined the influence of bioaugmentation on metal concentrations (aluminum, cadmium, chromium, cobalt, copper, iron, lead, manganese, molybdenum, nickel and zinc) in anaerobically digested sewage sludge. To improve the digestion efficiency, bioaugmentation with a mixture of wild-living Archaea and Bacteria (MAB) from Yellowstone National Park, USA, was used. The total concentration of all metals was higher in the digestate than in the feedstock. During anaerobic digestion, the percent increase in the concentration of most of metals was slightly higher in the bioaugmented runs than in the un-augmented runs, but these differences were not statistically significant. However, the percent increase in cadmium and cobalt concentration was significantly higher in the bioaugmented runs than in the un-augmented runs. At MAB doses of 9 and 13% v/v, cadmium concentration in the digestate was 211 and 308% higher than in the feedstock, respectively, and cobalt concentration was 138 and 165%, respectively. Bioaugmentation increased over 4 times the percentage of *Pseudomonas* sp. in the biomass that are able to efficiently accumulate metals by both extracellular adsorption and intracellular uptake. Biogas production was not affected by the increased metal concentrations. In conclusion, bioaugmentation increased the concentration of metals in dry sludge, which means that it could potentially have negative effects on the environment.

## Introduction

Currently, a major part of heavy metal emissions resulting from human activity enters into wastewater. The physical–chemical processes involved in wastewater treatment are responsible for accumulation of these metals in suspended-growth biomass (activated sludge). A typical metal content in sludge is about 0.5–2% on a dry weight basis [1] and may reach 4% [2]. However, this depends on many diverse factors, such as local conditions (e.g. type of industries in the region), wastewater quality, environmental factors and microbial structure of sludge, which depends on the applied method of treatment [3].

Heavy metals are defined as metals of a density above 5 g cm^–3^ [4], and some of them such as iron (Fe), cobalt (Co), copper (Cu), zinc (Zn), cadmium (Cd), nickel (Ni), molybdenum (Mo), chromium (Cr), and lead (Pb) are of great environmental significance. Aluminum (Al), despite its lower density, is commonly included in research of this group of metals due to its predicted toxicity [5]. The main concern with heavy metals is that they are stable, not biodegradable, and can accumulate to potentially toxic concentrations, also along the food chain [6]. High contents of these elements lead to reduced microbiological activity, which negatively affects both aerobic and anaerobic processes [7]. Moreover, this may cause the digester upset or failure and limit the application of sewage sludge as fertilizer or soil conditioner. However, many heavy metals categorized as trace elements (Co, Ni, Cu, Mn, Fe, Zn, Se and Mo) are part of central ligands of the essential enzymes that initiate several anaerobic processes as well as components of some bacterial nucleic acids; these elements are also required for the synthesis of vitamins [8–10].

Heavy metals may be involved in some crucial processes including precipitation (as sulfides, carbonates or hydroxides) and biosorption to the solid fraction (mainly biomass and microbial extracellular polymers), as well as formation of complexes in solution with intermediates and/or products generated during digestion [8, 11]. Thus, they occur in sewage sludge in various forms, but can differ in their speciation and mobility. The comprehensive review by Chen et al. [8] describes heavy metals, among the other toxicants inhibiting anaerobic digestion, and discusses their inhibition mechanism. The toxicity of heavy metals depends first of all on their form and total concentration − only the fraction that can be solubilized is toxic to microorganisms. It is believed that the failure of anaerobic digesters occurs when the concentration of free ions exceeds some threshold value, different for individual metals [12]. Importantly, the inhibitory effect may concern variable anaerobic microorganisms: acetogenic [13], acidogenic [14], methanogenic [15], and sulfate-reducing bacteria [16]. According to research by Chipasa [11], heavy metals tend to concentrate in the sludge during anaerobic digestion. Thus, disposal of metal-laden digestate represents a high environmental hazard, which effectively excludes uses in agricultural applications as well as soil reclamation. In view of this, the evaluation of digestate quality is of great practical significance. Moreover, the variability of metal concentrations during anaerobic digestion of sewage sludge should always be determined.

Recently, an interesting strategy that leads to improvement of several biological and chemical processes has been applied to both aerobic and anaerobic treatments. In this technique of bioaugmentation, enriched or mixed cultures of allochthonous or indigenous microorganisms are involved to enhance a required biological activity in the system investigated, and thus to improve the process performance [17]. It has been used more frequently in anaerobic digestion to enhance both process stability and biogas/methane yields [18] as well as to recover system efficiency at stress conditions [19]. Depending on the microorganisms/consortia involved, an increase of hydrolysis and acidogenesis could be achieved [20, 21]. Moreover, an accumulation of volatile fatty acids resulting from imbalance between acidogenic and methanogenic microorganisms did not occur [22]. Owing to the higher microbial diversity and abundance, the process failure could be avoided and methane production increased. Tale et al. [23, 24] noted recovery of organically overloaded digesters and found as much as a 120% increase in methane production using propionate degrading culture, whereas Nielsen et al. [25] achieved a 93% increase of methane yield in two-stage bioaugmented thermophilic system; however it sustained for only a limited time after inoculation. Using a commercial product containing selected strains of bacteria from genera *Bacillus*, *Pseudomonas* and *Actinomycetes*, it was possible to obtain a 29% higher methane yield from solids in wastewater treatment plant [26].

Bioaugmentation has also been applied to the degradation of specific organic substances [27], lipid-rich substrates [28, 29], keratin-rich waste [30], lignocellulosic matter [25, 31–34], as well as seed biomass [35]. Schauer-Gimenez et al. [36] and Li et al. [37] proposed this method to reduce the recovery time of digesters exposed to toxic events. Fotidis et al. [38, 39] suggested bioaugmentation for efficient anaerobic digestion of ammonia-rich waste through alleviation of ammonia toxicity effect. Li et al. [40] found it helpful for improving the digestion performance in the systems with feedstock of high C/N ratio. Other authors have applied bioaugmentation to accelerate digester start-up [41] as well as for odor reduction [26, 42]. The beneficial effects mentioned above indicate that the influence of bioaugmentation on anaerobic digestion of sewage sludge is worth investigating. The question is how to get the helpful microorganisms.

The microorganisms strains for bioaugmentation may be obtained by culturing in the laboratory combined with the strains selection, growing *in situ* using indigenous consortia or buying commercial products designed for specific applications [26, 36, 43]. Commercial products are easy to obtain, store and dose and thus are the most useful in practice. Some of them, in addition to selected strains of microorganisms, contain enzymes (lipase, protease, cellulase, hydrolase) needed to accelerate the decomposition of biopolymers as well as micronutrients essential for anaerobic digestion processes (Fe, Ni, Cu, Mn, Zn, Se, Mo). However, the composition of most commercial products is usually not known because of patent protection. There are also bioaugmentative products based on the wild-living microorganisms. Among them, the mixture of Bacteria and Archaea (MAB) from Yellowstone National Park, USA, is worth considering. Application of the microorganism from archaeal domain seems to be promising because of their adaptations to extreme habitats (assisted by the possession of unique cell wall types), including environments of high salt content, high temperature, low pH, and acute anoxia. The enzymes provided by Archaea result in higher reaction rates and reduce the contamination problems [44]. This was confirmed by the recent study of Lebiocka et al. [45] on the anaerobic digestion of sewage sludge. The authors reported a significant increase in the rate constant of biogas production along with the MAB dose despite the decrease in hydraulic retention time. The advantages of bioaugmentation contributed to more frequent application of this method in a technical scale. However, the environmental effect of its implementation to the existing digesters should also be examined. Thus far, the influence of bioaugmentation on digestate quality has not been evaluated. This subject is worth investigating, especially in the aspect of heavy metal concentration and the possible environmental risk related to the agricultural application of digestate. Importantly, the researchers involved in bioaugmentation studies did not consider all the possible effects of using this strategy, focusing more on biogas production and organic removal. Considering all the aspects of bioaugmentation (both benefits and hazards) is of great importance in the context of its implementation in full-scale systems. Thus, the proposed holistic approach constitutes a novel concept of bioaugmentation research. In the present study, the influence of bioaugmentation on total metal concentrations in digestate was examined for anaerobic digestion of sewage sludge. The presented results are the continuation of previously mentioned research [45]. The novel aspect of the work involved evaluating the effect of adding a mixture of wild the bacteria and Archaea from Yellowstone National Park, USA, on the metal contents in the digestate, and determining how it may influence the environment.

## Materials and methods

### Material characteristics

Sewage sludge that included residues from primary and secondary treatment was obtained from the Puławy municipal wastewater treatment plant (WWTP), Poland, operating at the flow rate of 13 000 m^3^ d^-1^. Primary sludge from the gravity thickener and waste sludge from the mechanical belt thickener were sampled once a week and transported to the laboratory in separate containers. Therein, sludge was mixed at the volume ratio of 60:40 (primary:waste sludge), recommended as beneficial for obtaining maximal biogas production, then homogenized, manually screened through a 3-mm screen and partitioned. The sludge samples were stored at 4°C in a laboratory fridge for a week at the longest. Sludge prepared in this manner (designated as SS) was used as material for the study. The main characteristics of SS during experiments was as follows (the average value ± standard deviation are given): chemical oxygen demand (COD) of 46 ± 6.3 g L^–1^, volatile fatty acids (VFA) of 838 ± 251 mg L^–1^, total solids (TS) of 40.1 ± 2.5 g kg^–1^, volatile solids (VS) of 31.0 ± 1.9 g kg^–1^, pH 6.8 ± 0.3 and alkalinity of 812 ± 100 mg L^–1^.

**Fig 1:**
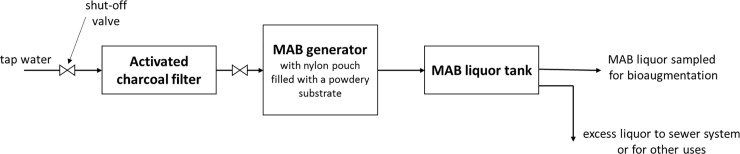
Scheme of MAB liquor preparation.

The mixture of Bacteria and Archaea used for bioaugmentation was provided by Soltech Co. the main representative of ArchaeaSolutions Inc. (Evansville, IN, USA) in Poland. The MAB liquor was prepared in continuous mode using a powdery substrate (interchangeable after 30 days), generator and the procedure given by ArchaeaSolutions Inc. (Evansville, IN, USA). The associated scheme is presented in Fig 1. The release of an appropriate microbial content of 1.08 g L^-1^ h^-1^ was achieved ensuring the flow rate of dechlorinated water through generator of about 0.5 L min^-1^ (Fig 1).The average TS and VS of the liquor were 0.48 and 0.042 g kg^–1^, respectively, and the COD value reached 22 g m^-3^. The VFA concentration of 21 g m^-3^, the alkalinity of 330 g CaCO_3_ m^-3^, and pH value of 7.16 were obtained. The microbial composition of the powdery substrate (ArcheaSolutions Inc.) after 1 day of cultivation at 37°C under constant mixing conditions in distilled water was investigated by next-generation sequencing (NGS), and sequencing results were deposited in the Sequence Read Archive (SRA, BioProject PRJNA431048). The sequencing was performed as described below (section Analytical methods). In the bioaugmenting mixture the most numerous genera were: *Methanosaeta* (32.8%), *Methanomethylovorans* (1.7%), *Methanobacterium* (1.7%), *Exiguobacterium* (16.9%), *Janthinobacterium* (12.1%), *Acinetobacter* (11.7%), *Stenotrophomonas* (10.6%), *Flavobacterium* (2.1%), *Brevundimonas* (1.9%) and *Herbaspirillum* (1.1%).

### Laboratory installation and operational set-up

**Fig 2:**
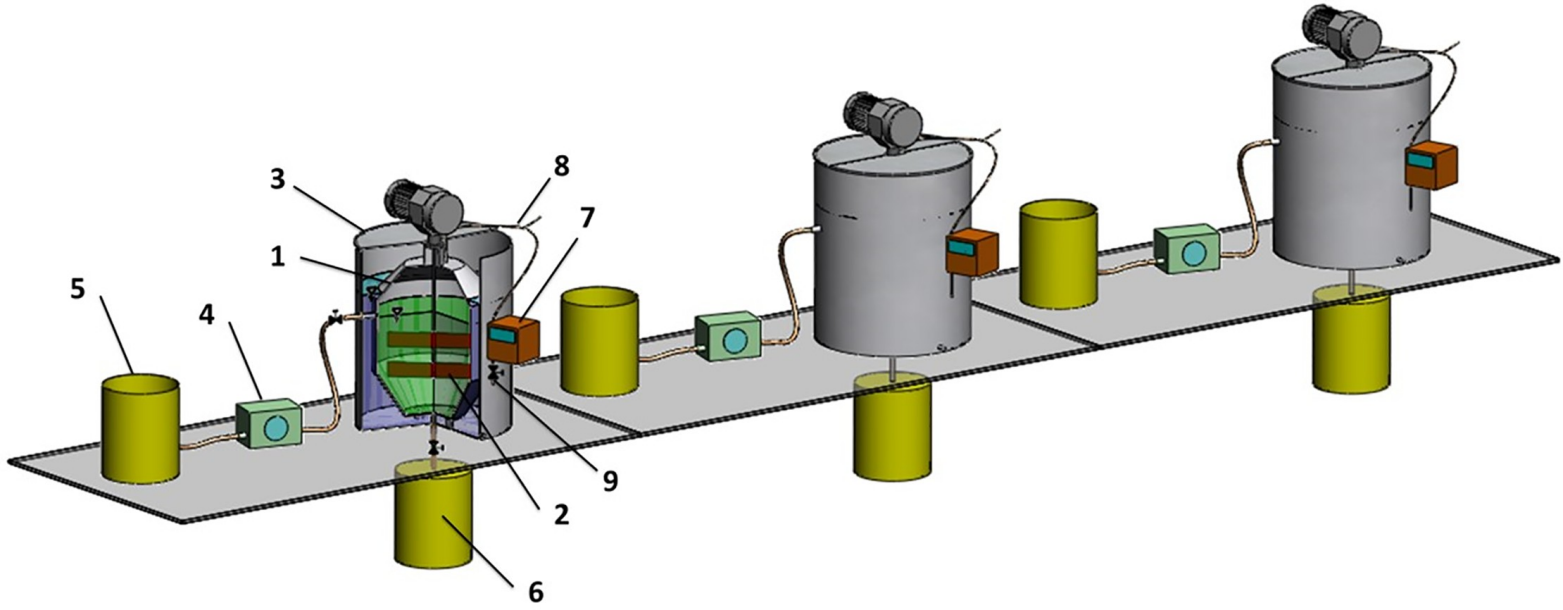
Laboratory installation used in the experiment. 1—anaerobic reactor, 2—mechanical stirrer, 3—heating jacket, 4—influent peristaltic pump, 5—influent storage vessel, 6—effluent storage vessel, 7—digital mass flow meter, 8—gaseous installation and gas sampler with a rubber septum, 9—dewatering valve.

The study was performed in anaerobic reactors operating at a temperature of 35°C in semi-flow mode. The laboratory installation consisted of three completely mixed digesters (each with an active volume of 40 L) working in parallel, equipped with a gaseous installation, an influent peristaltic pump and storage vessels. Feedstock was supplied to the upper part of the digester, and digestate was wasted through the bottom by gravity. The gas was taken up using a system consisting of pipelines linked with the pressure equalization unit and a mass flow meter. The scheme of the installation is shown in Fig 2.

Inoculum for the laboratory reactors was taken from the WWTP as a collected digestate from a mesophilic anaerobic digester. The adaptation of the digester biomass was achieved after 30 d.

Three runs were conducted, each lasting 90 days (d): 30 d for acclimatization and 60 d for measurements. The digesters were supplied once a day with an applied volume of SS or mixture of SS and MAB. In the first run (R1 –control) the reactor was fed with mixed sludge only. The second run (R2) and the third run (R3) were conducted to evaluate the influence of bioaugmentation on anaerobic digestion efficiency and digestate quality. The SS:MAB volumetric ratios were assumed on the basis of the results of batch experiments conducted previously by the authors. The main criterion was the enhanced biogas production efficiency as compared to the sole of SS anaerobic digestion, however retaining a suitable hydraulic loading rate of approximately 5% was also considered as in for semi-continuous systems. The detailed experimental settings are presented in Table 1.

**Table 1:** Experimental settings. (the mean OLR value and standard deviation are given).

Run	SS volume (L)	MAB volume (L)	SS:MAB volumetric ratio	OLR (kg VS m^-3^ day^-1^)	HRT (days)
**R1**	2.0	−	100:0	1.55 ± 0.06	20
**R2**	2.0	0.2	91:9	1.54 ± 0.05	18.2
**R3**	2.0	0.3	87:13	1.53 ± 0.05	17.4

It is widely known that in municipal wastewater treatment plants the HRT of 20 days is recommended for anaerobic digesters to ensure a highly efficient biogas production and organics removal. While introducing the MAB solution at the assumed doses, hydraulic loading rate was enhanced from 0.05 (R1) to 0.055 and 0.058 (for R2 and R3, respectively), because of an increase in the daily feedstock volume from 2 L to 2.2 and 2.3 L. Thus, the related HRT shortened from 20 days to 18.2 and 17.4 days.

### Analytical methods

TS and VS were determined according to the Standard Methods for the Examination of Water and Wastewater [46]. Analogously, the distinctive parameters (COD, VFA, alkalinity, and pH) were measured. The analysis was made once weekly in the feedstock and twice weekly in the digestate (for each reactor) during the entire cycle of measurements (60 d). VS removal (η_VS_) was evaluated based on organic matter decomposition.

The biogas production was measured using Aalborg (Orangeburg, NY, USA) digital mass flow meter. The biogas composition was determined using Trace GC-Ultra (Thermo Fisher Scientific, Milan, Italy) gas chromatograph coupled with a thermal conductivity detector (TCD) fitted with divinylbenzene (DVB) packed columns. The Rt-Q-Bond column was used for determination of CH_4_ and CO_2_ concentrations. The analysis was made using the following parameters: injector 50°C and detector 100°C. The carrier gas was helium with a flux rate of 1.5 cm^3^ min^-1^. Peak areas were determined by the computer integration program (CHROM_CARD).

Determination of total metal content, both in the feedstock that was supplied to the reactors and in the digestate was carried out using ICP-OES method (inductively coupled plasma optical emission spectrometry). Quantitative analysis was performed on a JY238 Ultrace (Jobin Yvon-Horriba, France) using an external calibration method following microwave digestion. The homogenized samples of 1 g were digested in an acid mixture of HNO_3_:HCl (5:2). The digestion was carried out for 45 min at 180^○^C and at a pressure of 1.8 MPa.

The concentrations of the following metals were determined: Al, Cd, Co, Cr, Cu, Fe, Mn, Mo, Ni, Pb and Zn. This was made at different emission bands, the wavelengths being as follows (in nm): Al– 308.215 and 394.401, Cd– 228.802, Co– 228.616, Cr– 267.716, Cu– 324.754, Fe– 259.940, Mn– 257.610, Mo– 202.030, Ni– 221.647, Pb– 220.353 and Zn– 213.856. Detection limits were established individually for each measurement series and were not higher than 10 μg L^-1^ for all metals.

The feedstock samples for metal content analysis were taken three times during the entire cycle of measurements. This was performed once weekly, on the same day for all reactors. The digestate was also sampled three times, considering that the hydraulic retention time (HRT) was adequate for specified runs. The measurements were made in triplicate for each sample and the results were presented as the mean of the nine measurements for each reactor.

### Statistical analysis

The statistical analysis of biogas production was accomplished via ANOVA involving Shapiro–Wilk’s, Levene’s and Tukey’s tests by the means of Statsoft Statistica software (version 10, TIBCO Software Inc., Palo Alto, CA, USA). The differences were considered as statistically significant at p < 0.05.

### Molecular analysis

Microbial structure of biomass from digesters was analyzed after 3 months of digester operation. The samples of biomass were stored at -20°C. Isolation of DNA was performed using GenElute™ Bacterial Genomic DNA Kit (Sigma-Aldrich, Germany). Integrity of DNA was confirmed by agarose electrophoresis while purity and concentration was measured using a Biophotometer (Eppendorf, Germany). Archaeal and bacterial 16S rDNA gene sequences were amplified using a universal 515F (GTGCCAGCMGCCGCGGTAA) and 806R (GGACTACHVGGGTWTCTAAT) primer set and then sequenced in Research and Testing Laboratory (USA) using the MiSeq Illumina platform. The obtained sequences were analyzed bioinformatically as described in Świątczak et al. [47]. Because samples had a similar number of reads normalization was not performed to avoid data loss. For rarefaction analysis a module of the RDPipeline was applied. The sequences have been deposited in SRA as BioProjectPRJNA380917 entitled ‘Metagenome of bioaugmented anaerobic digesters’.

## Results and discussion

The environmental impact analysis of the digested medium was performed by evaluating the changes of TS, VS and metal concentrations before and after digestion in bioaugmented and non-bioaugmented systems. In order to assess the possible toxic impact of metals on the anaerobic digestion process, the biogas yields, VS removal and methane concentration were determined (Table 2). More detailed information in the field of biogas production, kinetics and process stability is presented in the study performed by Lebiocka et al. [45].

**Table 2:** Biogas production, methane concentration, and VS removal for runs [45]. (the mean value and standard deviation are given).

Parameter	Unit	Run		
		R1	R2	R3
Biogas yield	m^3^ kgVS_added_^-1^	0.38 ± 0.07	0.40 ± 0.07	0.40 ± 0.07
CH_4_ concentration	%	56.3 ± 1.9	56.6 ± 1.6	56.2 ± 2.1
VS removal	%	46.7 ± 3.8	47.3 ± 4.1	49.2 ± 4.7

**Fig 3:**
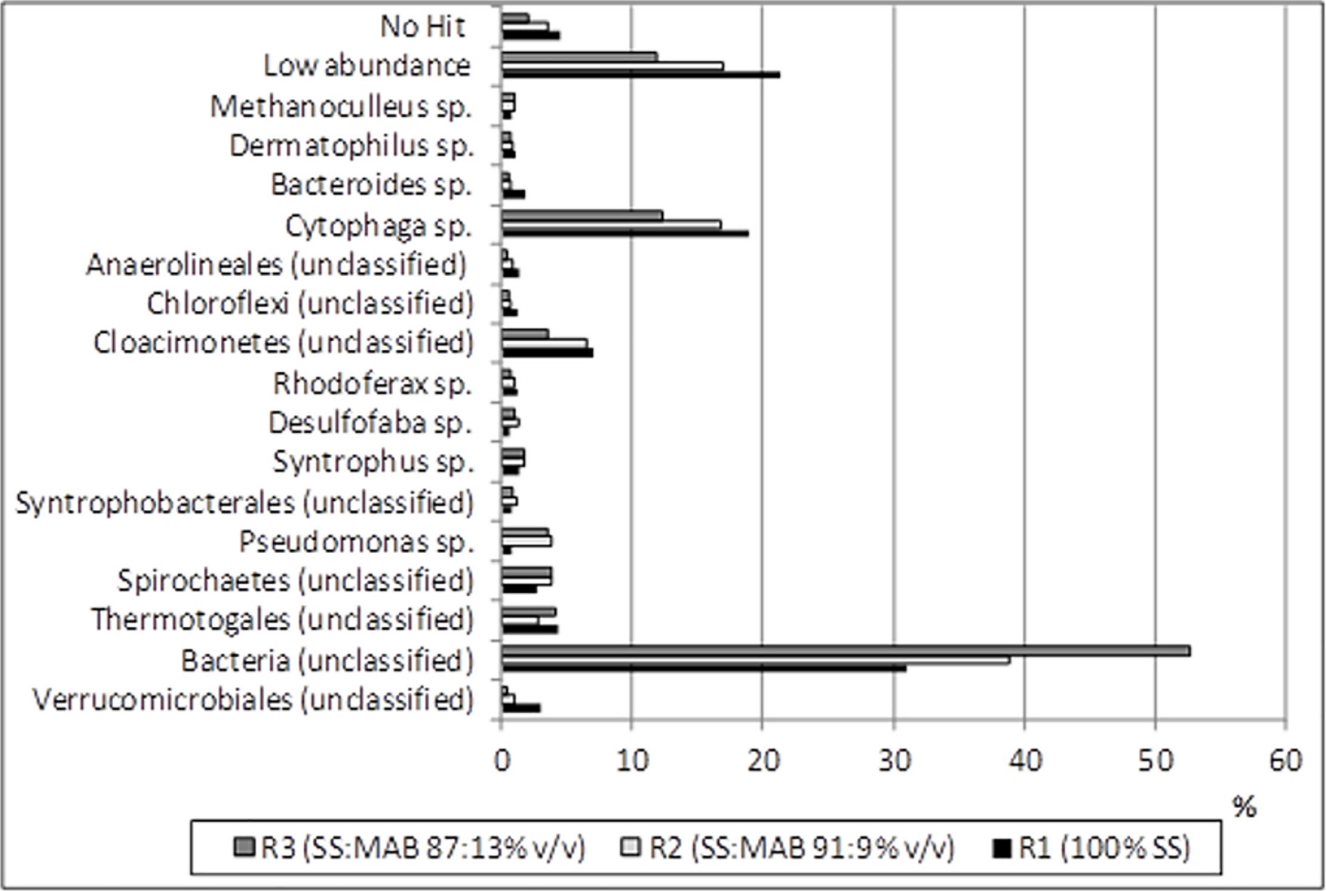
Percentage of archeal and bacterial taxa in biomass from the experimental reactors (in the table only taxa with abundance over 1.0% in one of the analyzed reactors were presented; the more detailed data are presented in Lebiocka et al. [45]).

The average TS were greatest in R1 (sewage sludge) both in feedstock and digestate: 40.1 and 24.0 g kg^–1^, respectively. In contrast, when feedstock was enriched by MAB there were correspondingly lower levels in R2 (36.4 and 21.9 g kg^–1^) and R3 (34.2 and 20.1 g kg^–1^). The same trend was observed for VS. The average values in feedstock were as follows: 31.0 (R1), 28.1 and 26.4 g kg^–1^ (R2 and R3) and in digestate VS reached 16.5, 15.0 and 13.5 g kg^–1^, respectively. Thus, the greater the MAB dose, the lower the average TS and VS content. This could probably be explained by dilution of the reactor’s influent with MAB as well as an effective decomposition of organic and inorganic matter to gaseous end products. Good decomposition of organic substrates may have resulted from the presence in all experimental reactors microorganisms belonging to Bacteroidetes (18.9–12.3%, Fig 3). Sewage sludge is rich in hard-to-degrade lignins, cellulose and hemicelluloses [48]. It was observed that bioaugmentation with an acetate-type fermentation bacterium belonging to Bacteroidetes increased the methane yield by 19–23% during corn straw digestion mostly due to increased removal rates of cellulose and hemicelluloses [21].

The bioaugmented anaerobic digestion of sewage sludge was more efficient (R2 and R3) compared to the control (R1). Despite the progressive decrease in HRT from 20 d (R1) through 18.2 d (R2) to 17.4 d (R3), the biogas yields were still similar (Table 2) (the differences of means were not statistically significant). Moreover, methane content was similar, independently of the MAB dose. The process was stable and the pH was maintained in the range typical for methanogenesis 7.72–7.99 (lower value for R3, upper for R1 and middle value of 7.86 for R2). The VS removal was higher despite the shortened HRT (Table 2) indicating that MAB improved the sludge digestion due to a better decomposition of organic substrates. This explanation is consistent with research by Duran et al. [26] regarding selected strains of the *Baccillus*, *Pseudomonas* and *Actinomycetes* species used for bioaugmentation.

As mentioned above, TS decreased with the amount of MAB added, both in feedstock and digestate. Considering weight loss during anaerobic digestion, an increment of total metal content in digestate could be expected–with the maximum for the bioaugmented run with the greatest dose of MAB (i.e. R3).

Determination of the average total concentration of metals both in the feedstock and digestate was performed for specified runs (Table 3).

**Table 3:** Average total concentration of metals and their total content on dry weight basis (± standard deviation) in feedstock and digestate.

Metal	Unit	R1		R2		*R3*	
		feedstock	digestate	feedstock	digestate	feedstock	digestate
Al	mg L^–1^	146.25 ± 17.75	148.94 ± 32.11	145.30 ± 14.99	152.54 ± 18.25	138.05 ± 16.32	157.76 ± 22.15
	mg kg TS^–1^	3660 ± 439	6270 ± 411	4001 ± 327	7121 ± 167	4386 ± 332	8025 ± 206
Cd	mg L^–1^	0.0430 ± 0.0140	0.0540 ± 0.0107	0.0401 ± 0.0124	0.0735 ± 0.0111	0.0420 ± 0.0136	0.1070 ± 0.0104
	mg kg TS^–1^	1.1 ± 0.4	2.3 ± 0.1	1.1 ± 0.3	3.4 ± 0.1	1.3 ± 0.3	5.4 ± 0.1
Co	mg L^–1^	0.0711 ± 0.0135	0.0908 ± 0.0088	0.0710 ± 0.0127	0.0998 ± 0.0140	0.0702 ± 0.0108	0.1166 ± 0.0096
	mg kg TS^–1^	1.8 ± 0.2	3.8 ± 0.1	2.0 ± 0.2	4.7 ± 0.1	2.2 ± 0.2	5.9 ± 0.1
Cr	mg L^–1^	0,3185 ± 0.1831	0,3320 ± 0.1276	0.3067 ± 0.1266	0.3375 ± 0.0956	0.3041 ± 0.1378	0.3520 ± 0.1144
	mg kg TS^–1^	8.0 ± 4.5	14.0 ± 1.6	8.5 ± 2.8	15.8 ± 1.0	9.7 ± 2.8	17.9 ± 1.1
Cu	mg L^–1^	1.7855 ± 0.1765	2.0846 ± 0.2389	1.6807 ± 0.1789	1.8989 ± 0.1621	1.4583 ± 0.1432	1.7943 ± 0.1526
	mg kg TS^–1^	44.7 ± 4.4	87.8 ± 3.1	46.4 ± 3.9	88.6 ± 1.5	46.3 ± 2.9	91.3 ± 4.1
Fe	mg L^–1^	89.52 ± 9.54	95.05 ± 20.13	82.26 ± 9.03	87.87 ± 20.07	80.69 ± 9.22	89.41 ± 22.95
	mg kg TS^–1^	2239 ± 236	4001 ± 258	2269 ± 197	4102 ± 184	2564 ± 188	4548 ± 214
Mn	mg L^–1^	5.43 ± 1.05	5.72 ± 1.14	5.14 ± 1.28	5.42 ± 0.97	4.50 ± 1.03	5.24 ± 0.88
	mg kg TS^–1^	136 ± 26	241 ± 15	142 ± 28	253 ± 9.0	143 ± 21	267 ± 8.0
Mo	mg L^–1^	0.0683 ± 0.0026	0.0704 ± 0.0020	0.0798 ± 0.0035	0.0831 ± 0.0021	0.0572 ± 0.0041	0.0639 ± 0.0030
	mg kg TS^–1^	1.7 ± 0.1	3.0 ± 0.05	2.2 ± 0.8	3.9 ± 0.1	1.8 ± 0.08	3.3 ± 0.03
Ni	mg L^–1^	0.1522 ± 0.0776	0.1573 ± 0.0998	0.1368 ± 0.0773	0.1411 ± 0.0819	0.1421 ± 0.0624	0.1577 ± 0.0619
	mg kg TS^–1^	3.8 ± 1.9	6.6 ± 1.3	3.8 ± 1.7	6.6 ± 0.8	4.5 ± 1.3	8.0 ± 0.6
Pb	mg L^–1^	0.4341 ± 0.0912	0.4461 ± 0.1287	0.4320 ± 0.1176	0.4419 ± 0.0823	0.4393 ± 0.1438	0.5004 ± 0.1165
	mg kg TS^–1^	10.4 ± 2.3	18.8 ± 1.7	11.9 ± 2.6	20.6 ± 0.8	14.0 ± 2.9	25.5 ± 1.1
Zn	mg L^–1^	14.41 ± 2.28	17.40 ± 4.18	13.87 ± 2.45	15.90 ± 5.03	11.73 ± 3.11	15.50 ± 6.27
	mg kg TS^–1^	360 ± 57	732 ± 54	383 ± 53	789 ± 46	373 ± 63	789 ± 58

The average total metal content on a dry weight basis (Table 3) was used for environmental impact analysis. This parameter allowed normalization and comparison of data from different runs.

On this basis, it can be estimated that the total concentration expressed in mg L^-1^was lower for most metals (Al, Cr, Cu, Fe, Mn and Zn) in bioaugmented feedstock than for non-bioaugmented sewage sludge (Table 3); moreover, concentrations decreased along with the increase of MAB dose (9 and 13% v/v, respectively). However, for some metals there were similar levels in all comparisons (Cd, Co and Pb) or no clear trend was observed (Mo and Ni). It was assumed that the addition of MAB probably diluted the sewage sludge (the lower TS value in bioaugmented runs seems to confirm this), thus the metal concentrations in feedstock decreased in most cases with the exception of Cd, Co and Pb. In contrast, there was an opposite trend on a dry weight basis. An increase of the total metal content in bioaugmented feedstock occurred (mostly related to a dose of MAB) due to the lower TS content.

The relative abundance of metals in sewage sludge samples before digestion followed the general order: Cd < Mo < Co < Ni < Cr < Pb < Cu < Mn < Zn < Fe < Al (Tables 1 and 2). A similar sequence was observed in samples enriched by MAB, except that there was less Co than Mo in R2.These results are consistent with other research focused on sewage sludge (11, 3); however, in these studies a small group of metals was investigated and their concentrations were one order of magnitude higher than in the present study (except for Cd and Pb). This may be due to the lack of high industrial loads in the supply to the Puławy WWTP, which was reflected in low levels of metals in sewage sludge. In contrast, Dong et al. [49] noted a different order in dewatered sewage sludge (with the use of a high-molecular weight flocculant based on polyacrylamide): Pb < Ni < Cu < Cr < Zn.

**Fig 4:**
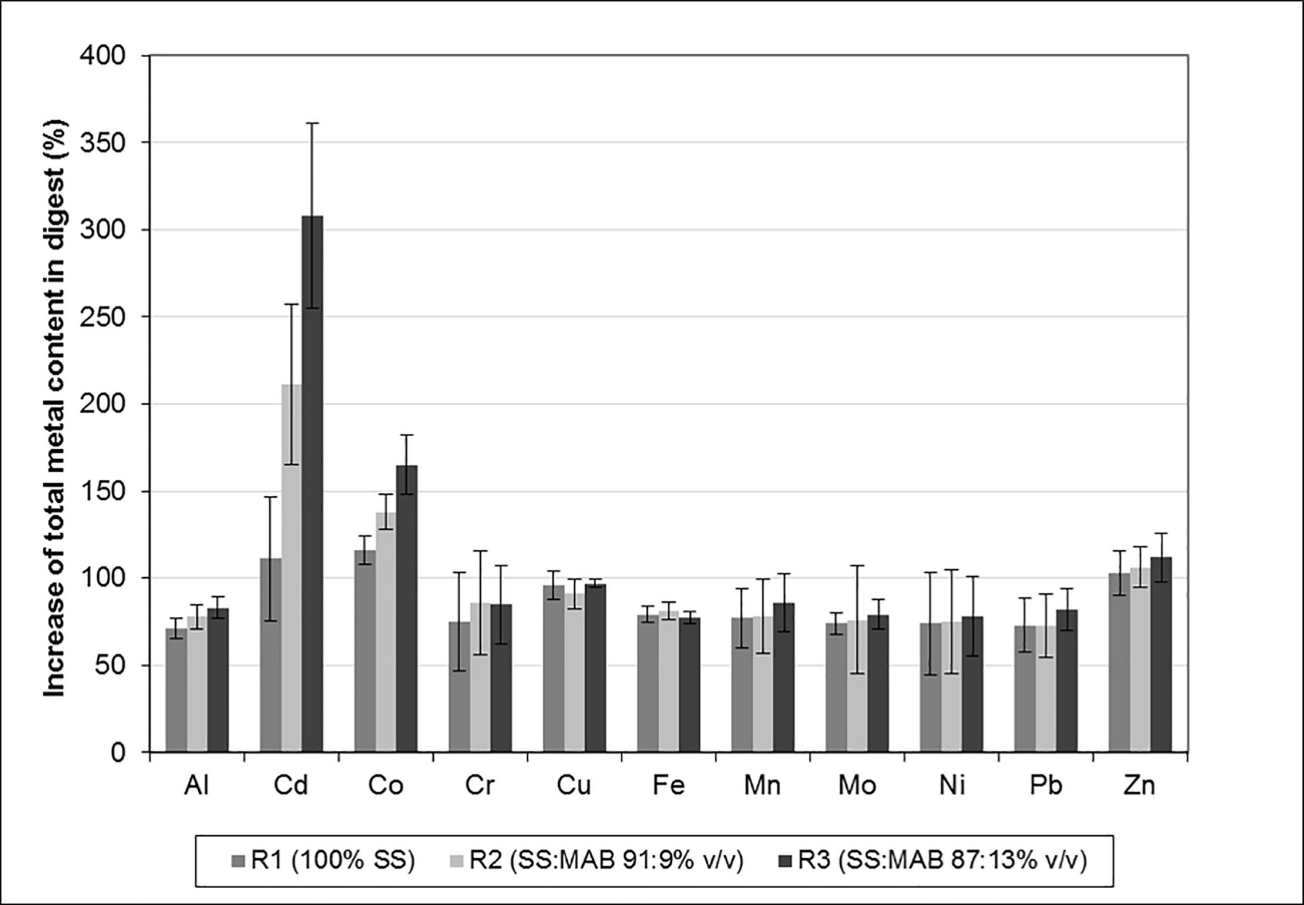
Increases of average total metal content in the digestate compared to the feedstock (%), error bars show 95% confidence interval for the mean.

The digested medium had an increased total content of all metals compared to the feedstock (Table 3). This observation is consistent with research on sewage sludge by Chipasa [11], who found that Cd, Cu, Pb and Zn concentrations in digestate exceeded the corresponding values in undigested sludge by 50–99%. In the present study, the corresponding increases were approximately 70–103% in R1, with the exception of 111 and 116% increases in the concentrations of Cd and Co, respectively (Fig 4). The pattern of increases was similar in the bioaugmented runs (i.e. R2 and R3), although the percentage increases for most metals (74–112%) were a little higher. Only the changes in Co and Cd concentrations differed significantly between reactors (one-way ANOVA); their concentrations exceeded the corresponding values in feedstock by 211 and 308% (Cd) and 138 and 165% (Co) in R2 and R3, respectively, and the size of the increase depended on the MAB dose (Fig 4).

**Fig 5:**
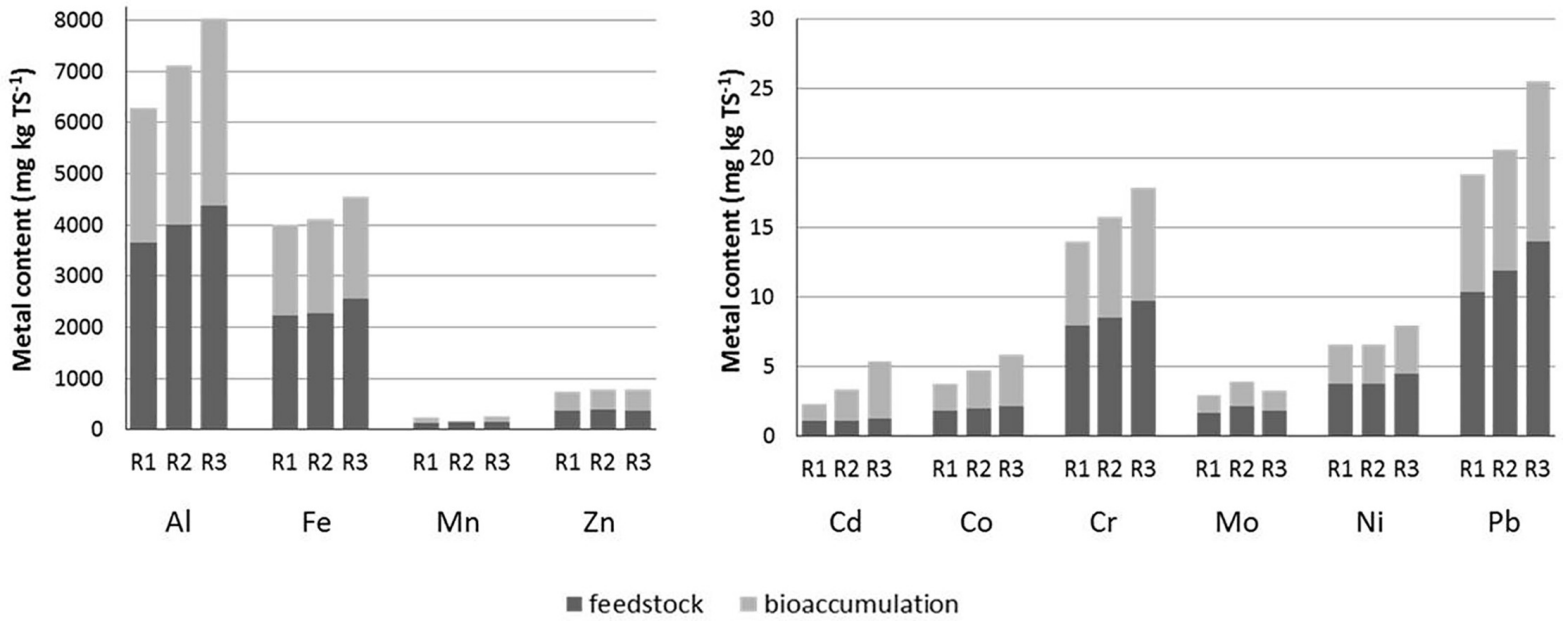
Mass balance of heavy metals in reactors, total height of the bar corresponds to the concentration of heavy metals in the digestate (average values are presented).

The mass balance for heavy metals showed their bioaccumulation throughout anaerobic digestion both in bioaugmented and un-augmented reactors (Fig 5). However, different levels of bioaccumulation were found with a spectacular difference for Cd and Co corresponding to the above-mentioned increases.

It should be noticed that in bioaugmented runs there was no association between the increases in metal concentration and the biogas yields, methane concentrations, and VS removal; thus the metals did not seem to have a toxic effect. This was in contrast to the results of the study by Abdel-Shafy and Mansour [50], who reported a significant decrease in both biogas production and VS removal, as well as an accumulation of organic acid intermediates resulted from Hg, Cd and Cr(III) toxicity in non-bioaugmented anaerobic digestion of sewage sludge. The above might indicate an immobilization of heavy metals in bioaugmented systems, resulting both from the differences in microbial selectivity and ability to take up metals. Metal remediation potential is strictly related to the selection of species in the bioaugmentation mixture. A study on removal of extractable metals such as Pb, Cu, and Al through bioaugmentation conducted with inocula of bacterial species isolated from leachate-contaminated soil has shown that the efficiency of metal removal was significantly lower when all isolated microbes were used than when only three species were used (*Lysinibacillus* sp., *Bacillus* sp., and *Rhodococcus* sp.) [51].

Bioaugmentation with even one group of bacteria involved in methane fermentation affects all steps of the process. Bioaugmentation of methane fermentation with hydrogen-producing acetogens (HPAs) obtained through enrichment and subculturing of anaerobic sludge with the use of butyric acid improved not only hydrogen-producing acetogenesis but also hydrolysis/acidogenesis and methanogenesis. At a bacterial number-to-activated sludge ratio of 1:9, inoculation with HPAs improved the yield and rate of methane production by a factor of 2.1 and 2.0, respectively [52]. In our study, bioaugmentation favored growth of *Pseudomonas* sp. that were not present in the MBA. The percentage of *Pseudomonas* sp. increased from 0.8% in the control reactor to over 3.5% in the bioaugmented digesters (Fig 3). In the same time increased Cd and Co accumulation was observed in the bioaugmented digesters. Previous studies indicated that *Pseudomonas* sp. may improve the uptake of heavy metals from the environment. During their active growth phase, *Pseudomonas aeruginosa* strain efficiently accumulated more than 75% of soluble cadmium (8 mM) from growth medium, and more than 89% from Cd-amended industrial wastewater; cadmium accumulated mostly in the membrane and periplasm of cells [53]. *Pseudomonas* sp. were also involved, along with *Brevundimonas* sp. and *Stenotrophomonas* sp., in nickel, cobalt and copper uptake from contaminated systems [54].

The primary mechanism of metal accumulation by microbes is biosorption followed by bioaccumulation into the cells, controlled siderophore production, enhanced respiration and modified protein profile [55, 56] *Pseudomonas halodenitrificans* efficiently biosorbed Co on its cell walls and the biosorption was significantly inhibited by the presence of divalent ions such as Ca [57]. *Pseudomonas* sp. exposed to Al shifts its metabolism toward the production of organic acids and lipids that play a vital role in chelating and immobilizing Al [58]. Low molecular weight ligands that chelate iron and other metals produced by microorganisms are called siderophores [59, 60]. The major siderophore produced by *Pseudomonas aeruginosa* is pyoverdine (PVD). Hannauer et al. [60] found that PVD-metal complexes could accumulate in the periplasm in different concentrations depending on the presence or absence of a specific efflux pump (PvdRT-OpmQ). Its absence resulted in higher levels of metals in the periplasm since the complexes were not expelled into the extracellular medium. With an inactive efflux pump, metal content increased as much as 1050% for Co^2+^ and 1005% for Zn^2+^, or as little as 45% for Cd^2+^ and 32% for Fe^3+^. It is worth noting that, in addition to changes in transport mechanisms, differences between microbial consortia may also lead to differences in metal accumulation. Investigation of protein response to Cd presence in *Pseudomonas aeruginosa* san ai cells indicated that almost a third of the total numbers of 60 proteins that were up-regulated in Cd-amended culture were metalloproteins. In the presence of Cd denitrification proteins were over expressed but not active, suggesting their protective role in conditions of heavy metal exposure [56].

Metals can also be accumulated by adsorption on extracellular polymeric substances that are excreted by bacteria. EPS have an important influence on metal binding capacity because of their high concentration of active sites, e.g. carboxyl groups [61]. *Pseudomonas* sp. can produce adhesive EPS, and this production increases as the length of incubation in starvation conditions is extended [62]. Moreover, it depends on the available source of organics. Production of EPS by *Pseudomonas aeruginosa* G1 and *Pseudomonas putida* G12 in media containing various amounts of glucose, mannose, fructose, and xylose was highest in the medium containing xylose. Maximum EPS yield was 368 mg L^-1^ with strain G1 cultivated in 3% (w/v) xylose [63]. *P*. *aeruginosa* are also able to produce biosurfactants such as rhamnolipids that complex heavy metals. Neilson et al. [64] evaluated rhamnolipid synthesis in the presence of heavy metals. Cd-induced *rhlB* expression was observed in mid-stationary phase (53 h) and sustained production of rhamnolipid was completed in 96-h late stationary growth phase. Presence of Cd also increased the ratio of dirhamnolipids to monorhamnolipids, which was favorable because the complexation constant for dirhamnolipid-Cd is several orders of magnitude larger than that of monorhamnolipid-Cd.

In our study, bioaugmentation increased the number of sequences that have only been identified at the kingdom level, indicating that many unknown species were present in the biomass and may have been involved in metal up-take.

The increase in content of a metal during digestion was not proportional to its initial content in the feedstock. This was consistent with research by Chipasa [11]. Although the relative abundance of metals after digestion was similar to that in undigested samples (only the relative abundance of Mo and Cd differed between bioaugmented and non-bioaugmented runs). Although bioaugmented and non-bioaugmented systems differed in terms of the percent increase of individual metals, these percent increases followed the same order in both systems (Fig 4). The results suggested that for the lowest total metal content in sludge before digestion there was the highest increase following the process. It was especially so regarding Cd and Co. However, introducing MAB led to much greater increases and the metals were much more concentrated in dry bioaugmented compared to non-bioaugmented sludge.

In principle, the contents of metals in bioaugmented and non-bioaugmented sludge after anaerobic digestion were in accordance with the compliance limits for agriculture land application [65]. However, since bioaugmentation increased concentration of metals on a dry weight basis, the use of bioaugmentation should be carefully considered for situations in which sewage sludge is rich in metals. In such an event, bioaugmentation could exclude the digestate from safe use in agricultural application or reclamation.

To sum up, the total metal content observed in the digestate tended to increase more in bioaugmented runs; moreover, the response to MAB was dose-dependent for most metals (the higher the dose, the more concentrated metals in the dry digested sludge). Thus, the addition of MAB could unfavorably affect the bioaugmented digestate quality.

## Conclusions

The digestate showed an increased total content of all metals compared to feedstock data. The percentage increase of most metals was slightly higher in bioaugmented compared to non-bioaugmented runs. There was a significant difference for Cd and Co; their concentrations exceeded the corresponding values in feedstock by 211 and 308% (Cd) and 138 and 165% (Co), respectively, accompanying an MAB dose. Bioaugmentation increased the percentage of *Pseudomonas* sp. that are able to efficiently accumulate metals by both extracellular adsorption and intracellular uptake. There was no coherency between the increments of metals and the biogas yields, thus their toxic impact seemed not to occur. Metals were more concentrated in dry sludge following bioaugmentation compared to non-bioaugmented process and the response to MAB was dose-dependent. Thus, this was found to potentially unfavorably affect the environment because of introducing the greater loads of metals. The results indicated that a holistic approach should be considered along with applying a bioaugmentation strategy.

## Supporting information

S1 Data (XLS)
